# Ocean warming favours a northern *Argyrosomus *species over its southern congener, whereas preliminary metabolic evidence suggests that hybridization may promote their adaptation

**DOI:** 10.1093/conphys/coad026

**Published:** 2023-07-03

**Authors:** Brett A Pringle, Murray I Duncan, Alexander C Winkler, Samuel Mafwila, Charmaine Jagger, Niall J McKeown, Paul W Shaw, Romina Henriques, Warren M Potts

**Affiliations:** Department of Ichthyology and Fisheries Science, Rhodes University, Makhanda, South Africa; Advance Africa Management Services, Johannesburg, South Africa; Department of Ichthyology and Fisheries Science, Rhodes University, Makhanda, South Africa; South African Institute for Aquatic Biodiversity, Makhanda, South Africa; University of Seychelles and Blue Economy Research Institute, Anse Royale, Mahe, Seychelles; Department of Ichthyology and Fisheries Science, Rhodes University, Makhanda, South Africa; Department of Fisheries and Aquatic Sciences, Sam Nujoma Campus, University of Namibia, Henties Bay, Namibia; Department of Fisheries and Aquatic Sciences, Sam Nujoma Campus, University of Namibia, Henties Bay, Namibia; Ministry of Fisheries and Marine Resources, Swakopmund, Namibia; Institute of Biological, Environmental and Rural Sciences, Aberystwyth University, Aberystwyth, UK; Institute of Biological, Environmental and Rural Sciences, Aberystwyth University, Aberystwyth, UK; Marine Genomics Group, Department of Biochemistry, Genetics and Microbiology, University of Pretoria, Pretoria, South Africa; Department of Ichthyology and Fisheries Science, Rhodes University, Makhanda, South Africa; South African Institute for Aquatic Biodiversity, Makhanda, South Africa

**Keywords:** physiology, ocean warming, northern Benguela

## Abstract

Anthropogenic-induced climate change is having profound impacts on aquatic ecosystems, and the resilience of fish populations will be determined by their response to these impacts. The northern Namibian coast is an ocean warming hotspot, with temperatures rising faster than the global average. The rapid warming in Namibia has had considerable impacts on marine fauna, such as the southern extension of the distribution of *Argyrosomus coronus* from southern Angola into northern Namibian waters, where it now overlaps and hybridizes with the closely related Namibian species, *A. inodorus*. Understanding how these species (and their hybrids) perform at current and future temperatures is vital to optimize adaptive management for *Argyrosomus* species. Intermittent flow-through respirometry was used to quantify standard and maximum metabolic rates for *Argyrosomus* individuals across a range of temperatures. The modelled aerobic scope (AS) of *A. inodorus* was notably higher at cooler temperatures (12, 15, 18 and 21°C) compared with that of *A. coronus*, whereas the AS was similar at 24°C. Although only five hybrids were detected and three modelled, their AS was in the upper bounds of the models at 15, 18 and 24°C. These findings suggest that the warming conditions in northern Namibia may increasingly favour *A. coronus* and promote the poleward movement of the leading edge of their southern distribution. In contrast, the poor aerobic performance of both species at cold temperatures (12°C) suggests that the cold water associated with the permanent Lüderitz Upwelling Cell in the south may constrain both species to central Namibia. This is most concerning for *A. inodorus* because it may be subjected to a considerable coastal squeeze.

## Teaser

This study used metabolic experiments to show that ocean warming in southern Angola and northern Namibia will likely favour Argyrosomus coronus over its cooler water relative, A. inodorus. Hybrids of the two species seem to be competitive and hybridization may provide opportunities for physiological adaptation to climate change.

## Introduction

Marine organisms are particularly vulnerable to climate-related disturbances because they may live relatively close to their environmental limits and the ocean lacks spatial refuges to avoid increasingly extreme environmental events (Howes *et al.,* 2015; Oliver *et al.,* 2018; Pinsky *et al.,* 2019). Without many environmental refugia, marine fishes are directly exposed to increasing levels of environmental variability, including temperature (Bates *et al.,* 2018; Duncan *et al.,* 2019), ocean acidification (Edworthy *et al.,* 2018) and low oxygen zones (Crear *et al.,* 2020). Predicting resilience in these future environmental conditions is challenging because the responses of species to changing environments are variable (Somero, 2010; Fulton, 2011), but critically important to develop appropriate management and adaptative strategies.

Because most fish are ectotherms, their physiological processes are governed by their external environment (Brown *et al.,* 2004), of which temperature is a primary driver (Bozinovic and Pörtner, 2015; Horodysky *et al.,* 2015; Schulte, 2015; Pinsky *et al.,* 2019). Whereas the response of fishes to increasing temperatures may vary (Gräns *et al.,* 2014; Audzijonyte *et al.,* 2016; Barneche *et al.,* 2019), changes in temperature regimes can influence their reproductive scope (Potts *et al.,* 2014a), life history characteristics (Pankhurst and Munday, 2011; Watson *et al.,* 2018), behaviour and distribution (Somero, 2002; Ling *et al.,* 2009; Brander, 2010; Pörtner, 2010) and their vulnerability to disease or predation (Arend *et al.,* 2011; Lapointe *et al.,* 2014). However, the response of a species may not be uniform throughout its distribution because the temperature change may be beneficial, insignificant, or deleterious depending on the area and direction of change. In warming conditions, individuals found near the warm edge of the distribution of the species are often most susceptible because they are often near the edges of their physiological tolerance (Wiens, 2016). In contrast, warming at the cold edge of marine systems may result in increases in the abundance of fish populations and range expansion into these regions (Wiens, 2016; Fredston-Hermann *et al.,* 2020).

There is an urgent need to predict the potential impacts of climate change on fishes and this has typically been done by correlating environmental variables with historical population responses (Horodysky *et al.,* 2015). Although this approach has been used extensively, understanding the physiological mechanisms underpinning these responses is necessary to refine predictive models (Deutsch *et al.,* 2015; Horodysky *et al.,* 2015; McKenzie *et al.,* 2016). Energy to execute performance-related processes is allocated from an aerobic energy budget, therefore, physiology experiments that quantify the rates of aerobic metabolism change in response to temperature variability can be used as a forecasting tool for how future temperature changes may constrain performance (Wikelski and Cooke, 2006; Urban *et al.,* 2016). When mechanistic models are parameterized with physiological predictive models for a range of populations or species, it may be possible to identify future resilient or vulnerable characteristics (Evans *et al.,* 2015; Horodysky *et al.,* 2015; Crear *et al.,* 2020) in a particular region and to implement management strategies to adapt to these changes (Huey *et al.,* 2012; Cooke *et al.,* 2013; McKenzie *et al.,* 2016).

Despite the incorporation of physiology metrics, mechanistic models do not consider the effects of adaptation and transgenerational acclimation (Munday, 2014). Acclimation to novel conditions can occur across generations when the offspring’s performance in an environment is enhanced because their parents have experienced the same environment (Munday, 2014). However, there are limits to the extent of the phenotypic change using this mechanism. In contrast to acclimation, adaptation can drive major shifts in the phenotypes of a species through natural selection. However, this is generally considered to be a relatively slow process, requiring multiple generations and may be too slow to facilitate adaptation to the rapidly changing ocean conditions experienced by many marine fishes.

Adaptation can however be accelerated through mechanisms, such as genetic drift (chance occurrence, such as a mass mortality event) or gene flow (movement of alleles into or out of a population through the migration of individuals) (D'Anatro and Lessa, 2011; Wang *et al.,* 2013). In cases where the movement of individuals places them in contact with closely related species, hybridization (interbreeding of two distinguishable populations (Harrison and Harrison, 1993)) may occur, and this may be a catalyst for rapid evolutionary change (Arnold *et al.,* 2012). Hybridization can also be considered beneficial in the face of climate change by introducing genetic variation to a population, which may increase the evolutionary potential of a population to a changing environment (Grant and Grant, 2010; Hoffmann and Sgrό, 2011).

The measurement of aerobic metabolic rate is becoming increasingly popular to assess the physiological consequences of fishes to a changing climate (Clark *et al.,* 2013). This is because warming affects biochemical reactions and metabolic demand, which can limit excess metabolic capacity and alter life history characteristics (Nelson, 2016; Causey *et al.,* 2019; Little *et al.,* 2020). Changes in temperature can therefore influence the energy available for performing fitness-related processes like growth, feeding and reproduction (Clark *et al.,* 2013; Little *et al.,* 2020).

The concept of aerobic scope (AS) posits that the available energy budget for various energetic processes (such as growth and reproduction) varies across environmental gradients (such as temperature) and is estimated as the difference between the maximum metabolic rate (MMR) and standard metabolic rate (SMR) (Fry, 1947, 1971; Claireaux and Lefrançois, 2007; Clark *et al.,* 2013; Pörtner *et al.,* 2017). When the AS is plotted against temperature, the resulting AS curve can be used to quantify the effects of temperature change on the free energy of fish, and although there are limitations to the concept, it is considered one of the most practical (Horodysky *et al.,* 2015). Despite the broad use of the AS concept to predict population-level responses to climate change, it is well recognized that there may be considerable intrapopulation (Metcalfe *et al.,* 2016; Crear *et al.,* 2019) and interpopulation variability.

Ocean warming hotspots (Hobday and Pecl, 2014) are critical research areas because they are early warning laboratories for climate change impacts (Pecl *et al.,* 2014). Research in these areas is not only necessary to facilitate rapid adaptation to change, but also offers the opportunity to validate methods, assess the accuracy of predictions and observe the early response of fishes to ocean warming (Hobday and Pecl, 2014). The coastal area between southern Angola and northern Namibia has been identified as an ocean warming hotspot where temperatures are rising at approximately 10 times the global average causing several climate-driven changes on regional marine fauna (Hobday and Pecl, 2014; Potts *et al.,* 2014b). One change is a poleward distributional shift of the coastal sciaenid, *Argyrosomus coronus* (Griffiths and Heemstra, 1995) from southern Angola into Namibia (Potts *et al.,* 2014a). The southward shift of *A. coronus* resulted in an increasing distributional overlap and hybridization with its closely related congener, *A. inodorus* (Griffiths and Heemstra, 1995)*,* an important Namibian coastal fishery species (Potts *et al.,* 2014b).

**Figure 1:**
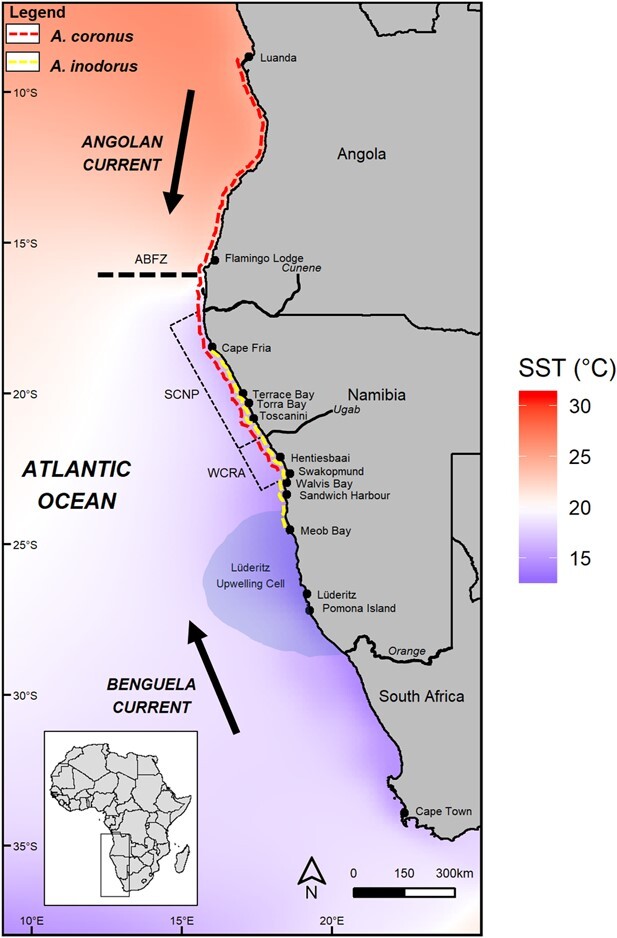
The west coast of southern Africa showing the known distribution of *Argyrosomus coronus (red)* and the Namibian population of *A. inodorus (yellow)*, with the region’s key oceanographic features and current mean SST. The combination of warm events as a consequence of Benguela Niños and local wind anomalies (Richter *et al.,* 2010), with the increased warming and southward movement of the ABFZ as well as the intensification of the cold Lüderitz Upwelling Cell, will likely increase SST variability in this region and create a highly variable ocean environment off the coast of central and northern Namibia (Potts *et al.,* 2014b). ABFZ, Angola Benguela Frontal Zone.

*Argyrosomus coronus* is a coastal predatory species distributed from central Namibia to northern Angola (Potts *et al.,* 2010) (red dotted line, Fig. 1), whereas *A. inodorus* is a southeastern Atlantic endemic and has several separate stocks in the region (Griffiths and Heemstra, 1995). The Namibian population of *A. inodorus* occurs only in central (from Meob Bay) and northern Namibia (up to Cape Fria) (Kirchner and Holtzhausen, 2001) (yellow dotted line, Fig. 1). Despite the morphological similarity (Supplementary Fig. S1), the habitat and life history characteristics of these two species are markedly different (Supplementary Table S1). The Namibian *A. inodorus* stock is primarily found in the nearshore and is likely restricted by an anoxic zone found at depths >20 m (Griffiths, 1997). This anoxic zone seems to force both west coast *Argyrosomus* species together into the shallow water habitat. The southward distribution of the Namibian population of *A. inodorus* is limited by the Lüderitz Upwelling Cell (Henriques *et al.,* 2015), whereas the northern distribution is limited by the warm water in northern Namibia (Fig. 1). It is hypothesized that these barriers limit further distribution of temperate inshore fishes due to a reduced metabolic potential in the warmer water temperatures in the north and the cooler water temperatures in the south.

Historical data (Supplementary Table S2) suggest that *A. inodorus* was the dominant species (~90%) of the recreational *Argyrosomus* catch in the West Coast Recreational Area (WCRA) and the Skeleton Coast National Park (SCNP) in Namibia between 1993 and 1995 (Kirchner, 1999; Holtzhausen *et al.,* 2001). There was no evidence of hybridization between the two species during this time (Van der Bank and Kirchner, 1997; Potts *et al.,* 2014b). However, catch data from 2008 to 2009 suggest that *A. coronus* outnumbered *A. inodorus* in the WCRA (Potts *et al.,* 2014b) (Supplementary Table S2), with the proportion of *A. coronus* in the fishery at 57%. Interestingly, the mtDNA and nuclear microsatellite DNA analyses of samples collected in 2009 identified ~3% of the individuals in the same region as hybrids (Potts *et al.,* 2014b).

Understanding the future overlap of these species and the physiological consequences of their hybridization is not only critical for the prediction and improved management of a fishery with newly arriving species (Frusher *et al.,* 2014; Maxwell *et al.,* 2015; Pecl *et al.,* 2017), but will also enhance our understanding of the response of fishes to warming. This will allow a proactive management approach that rapidly adjusts fisheries regulations as appropriate to maintain the sustainability of newly arrived species (Frusher *et al.,* 2014; Maxwell *et al.,* 2015; Pecl *et al.,* 2017). Therefore, this study aimed to compare the aerobic metabolic rate and AS curves of *A. inodorus* and *A. coronus* to better understand the likely response of these fishes to coastal warming. Although ocean warming-driven hybridization has been documented in marine fishes (Potts *et al.,* 2014b; Takahashi *et al.,* 2017; Takahashi *et al.,* 2021), there is little information on its physiological consequences and thus limited scope for exploring how to incorporate these metrics into mechanistic models or the development of species distribution models that account for adaptation (*e.g.* ΔTraitSDMs, Benito Garzón *et al.,* 2019). Accordingly, we also aimed to characterize the aerobic metabolic rate and AS of putative hybrids of the two species to gain a preliminary understanding of the potential consequences of hybridization on the physiological response of fishes to climate change. We hypothesized that the optimal temperature would be warmer for *A. coronus* than *A. inodorus* and that the hybrids of the two species would have a wider AS.

## Materials and Methods

### Ethics

All research activities were conducted under the Rhodes University Animal Research Ethics Committee (RU-AREC), reference number: 2019–0174-271, the Namibian Ministry of Environment and Tourism and the National Commission on Research Science and Technology, authorization number: 20190204.

### Fish collection and husbandry

*Argyrosomus coronus* and *A. inodorus* specimens were collected from the shore in the vicinity of the Toscanini old diamond mine in the SCNP, Namibia, between 2 March and 6 June 2019 (Fig. 1). Seventy-four individuals were caught using conventional marine shore-based angling tackle (12- to 15-ft graphite rods, fixed or revolving spool reels, nylon or braided lines with circle and J hooks) and transported in 1000- or 500-L tanks filled with oxygenated seawater to the Sam Nujoma Marine and Coastal Resources Research Centre (SANUMARC) mariculture laboratory in Hentiesbaai, Namibia. Fish were held in 13 800-L recirculating holding tanks, under natural light conditions, where mechanical and biofiltration and routine water changes were used to maintain the water quality and ambient water temperature (mean = 18.29°C, SD = 1.11°C). Fish were acclimated for a minimum of 4 weeks before experimentation and fed sardine *Sardinops ocellatus* and round sardinella *Sardinella aureti* three to four times per week*.*

### Experimental protocol

Fish were fasted for 36 h before experimentation. After fasting, fish were netted and individually placed in one of four custom-built respirometers [small: 14 cm internal diameter (ID) and 50 cm length (L); medium: 19 cm ID and 75 cm L; large: 24 cm ID and 100 cm L] to accommodate the varied size range of fish [31.5–72.5 cm total length (TL) in this study], at the same temperature of the holding tanks on the day, followed by a 12-h acclimation period, during which water temperature was adjusted (0.5°C per 45 min) to one of the test temperatures: 12 (*A. inodorus* n = 6; *A. coronus* n = 3), 15 (*A. inodorus* n = 6; *A. coronus* n = 3; hybrids n = 1), 18 (*A. inodorus* n = 9; *A. coronus* n = 3; hybrids n = 1), 21 (*A. inodorus* n = 8; *A. coronus* n = 3) or 24 (*A. inodorus* n = 8; *A. coronus* n = 2; hybrids n = 1) °C. The experimental test temperatures in this study were chosen because they represent the current and predicted future thermal conditions along the central Namibian coast (Junker *et al.,* 2017). The lowest test temperature was set at 12°C because the Lüderitz Upwelling Cell at the southern edge of *A. inodorus’* distribution is typically 11–14°C (Griffiths and Heemstra, 1995; Griffiths, 1997; Kirchner and Holtzhausen, 2001; Potts *et al.,* 2014b), whereas the upper test temperature was set at 24°C because the region’s water temperature is predicted to increase by ~2°C from its current average of 22°C in the summer months by 2050 (Hobday and Pecl, 2014; Potts *et al.,* 2014b; Potts *et al.,* 2018).

Oxygen consumption was measured in milligrams per Liter. Details of the intermittent respirometry experimental protocol as suggested by Killen *et al.* (2021) are outlined in Supplementary Table S6. After the 12-hour acclimation period, SMR (mgO_2_·L^−1^·min^−1^·kg^-b^) measurements were recorded using intermittent-flow respirometry (5-min measure; 15-min flush), for a minimum of 22 h to account for any diel variability (Chabot *et al.,* 2016). After SMR measurements, fish were individually removed from the respirometers, placed in a cylindrical tank and subjected to 3 min of chasing and tail grabbing to induce burst swimming, ensuring they became lethargic and non-responsive, and this was followed by exposure to air for 1 min (Clark *et al.,* 2013; Roche *et al.,* 2013; Norin and Clark, 2016) before being placed back into a sealed respirometer (within 20 s). The MMR (mgO_2_·L^−1^·min^−1^·kg^-b^) was estimated from the single steepest decline in oxygen concentration during 10 h of intermittent-flow respirometry after this chasing protocol. Experimental fish were removed after this 10-h period, and a 3-h blank was run to measure and account for background microbial respiration in the empty respirometers (Clark *et al.,* 2013; Svendsen *et al.,* 2016). Experimental fish were weighed (g) and measured (cm, TL). Fin clip and muscle tissue samples were removed from individual fish and stored in 95% ethanol for mtDNA sequencing to confirm the species/hybrid status of each individual.

### Genetic confirmation

Total DNA was extracted using a standard CTAB-chloroform/isoamylalcohol method (Winnepenninckx *et al.,* 1993). Each individual was genotyped at a fragment of the mitochondrial DNA (mtDNA) cytochrome oxidase I (COI) gene using the FF2d and FF1d primers described by Ivanova *et al.* (2007) and at five microsatellite loci (UBA5, UBA40, UBA91, UBA853, UBA854) developed by Archangi *et al.* (2009) and shown to be useful for hybrid identification in *Argyrosomus* (Potts *et al.,* 2014a) (Supplementary Table S3). Polymerase chain reactions (PCR) for each primer pair were performed in 10-μl volumes using a thermoprofile comprising 95°C for 3 min followed by 35 cycles of 95°C for 30 s, 50°C for 30 s and 72°C for 30 s, after which there was a final extension (72°C for 180 s) and cooldown (4°C for 60 s). Sequencing of mtDNA amplicons was performed using the FF2d primer with Big Dye technology and an ABI 3500 DNA analyser. The ABI 3500 analyser was also used to separate microsatellite alleles with genotypes inferred using PEAKSCANNER software.

The mtDNA sequences were edited and aligned using BIOEDIT (Hall, 1999) and all subsequent analyses were performed in ARLEQUIN 3.1 (Excoffier *et al.,* 2005) unless stated otherwise. Genetic variation was described using indices of haplotype and nucleotide diversity (*h* and π, respectively). A minimum spanning network was constructed in NETWORK (www.fluxus-engineering.com/sharenet.htm).

Microsatellite data were used to identify putative hybrids in the dataset, based on the posterior probability of assignment (*q*) for all individuals, following the protocol of Potts *et al.* (2014a). In brief, admixture was investigated using STRUCTURE and NewHybrids. STRUCTURE analyses were performed using three different models: 1) admixture model with independent allele frequencies; 2) admixture model with correlated allele frequencies and 3) non-admixture model with independent allele frequencies, as suggested by Henriques *et al.* (2016). Five independent analyses were run for two clusters (k = 2), for 500 000 MCMC steps for burn-in, followed by 2 000 000 MCMC steps. A threshold of *q* = 0.15 was chosen to separate parental individuals and putative hybrids (Potts *et al.,* 2014a). In addition, NewHybrids (Anderson and Thompson, 2002) was used to corroborate the results obtained with STRUCTURE. In this case, the threshold of posterior probability was set to *q* = 0.75 (Potts *et al.,* 2014b), using a Jeffreys before mixing and allele frequencies, and ran for 2 000 000 MCMC iterations. Putative hybrids were identified as individuals that were assigned as hybrids by both STRUCTURE and NewHybrids.

To ascertain that the microsatellite had enough power to detect hybrids, simulation studies were conducted. Fifty hybrids per hybrid class (F1, F2, backcross to *A. coronus* and backcross to *A. inodorus*) were generated in HYBRIDLAB (Nielsen *et al.,* 2006) and run through STRUCTURE and NewHybrids, using the same parameters as before, to assess resolution and accuracy (Supplementary Table S4).

### Data preparation

Oxygen consumption rates (*R*O_2_) for each measurement period (Supplementary Fig. S2) were quantified from the slope of the linear decline in oxygen following equation 1 (adapted from Svendsen *et al.,* 2016). Oxygen consumption rates with a coefficient of determination (R^2^) of <0.9 were omitted from the analysis. The first minute of the measurement period was considered a wait period and was not included in the linear regression.

Equation 1 \begin{align*} {RO}_2&=\left(\left({V}_{re}-M\right)\left(\frac{\Delta \left[{O}_{2a}\right]}{\Delta t}\times 60\right)\right)\notag \\&\quad-\left(\left({\mathrm{V}}_{re}-M\right)\left(\frac{\Delta \left[{O}_{2b}\right]}{\Delta t}\times 60\right)\left(\frac{V_{re}}{V_{re}-M}\right)\right) \end{align*}

*V_re_* is the total volume of the respirometer in liters, *M* is the mass of the specimen in kilograms expressed as l, Δ[O*_2a_*]/Δ*t* is the slope of the linear decrease in oxygen concentration during the measurement period and Δ[O*_2b_*]/Δ*t* is the slope of the linear decrease in oxygen concentration when no specimen was in the chamber (background respiration).

For each individual specimen, all metabolic rates that passed our quality threshold were grouped together and SMR was estimated as the 0.2 quantile, following the guidelines of Chabot *et al.* (2016). Maximum metabolic rate was estimated as the single highest rate of oxygen consumption following the exhaustive protocol (Killen *et al.,* 2017).

Before modelling the temperature dependence of metabolic rate data, we mass-corrected the data because a large size range was used in the experiment (454–2875 g). We first removed the effect of temperature on metabolic rate data by dividing with an Arrhenius function (Deutsch *et al.,* 2015) (Equation 2):

Equation 2 \begin{equation*} {RO}_{2\left( temp\ standardized\right)}={RO}_2\times \frac{-E}{e\ kT} \end{equation*}

where *E* is the average activation energy of ectotherms ~0.63 eV (Gillooly *et al.,* 2001), *k* is the Boltzmann constant 8.6173303 × 10^−5^ eV·K^−1^ and *T* is the absolute temperature in Kelvin.

We estimated the allometric mass scaling exponents for SMR and MMR (α) as the slope of the linear relationship between the natural logarithm of *R*O_2_ (temperature standardized) and the natural logarithm of mass (Deutsch *et al.,* 2015).

*R*O_2_ data were then converted into mass-specific metabolic rate measurements (*M*O_2_) by dividing with mass raised to the species scaling exponent following Equation 3:

Equation 3 \begin{equation*} {MO}_2=\frac{RO_2}{M^{\mathrm{\alpha}}} \end{equation*}

Where *M*O_2_ is the mass-specific SMR or MMR, *R*O_2_ is whole organism standard or maximum oxygen consumption rate, *M* is the mass of the organism (g) and α is the allometric mass scaling exponent.

Absolute AS was calculated using mass-specific metabolic rates using Equation 4:

Equation 4 \begin{equation*} AS= MMR- SMR \end{equation*}

### Statistical analysis

Standard metabolic rate—temperature relationships were modelled with an Arrhenius function described in equation 5 (Gillooly *et al.,* 2001), where $a$ is the rate coefficient, *E* is the activation energy, k is the Boltzmann’s constant (eV) and T is the absolute temperature in Kelvin. We estimated the constants of the Arrhenius models; $a$ and E as the back-transformed intercept and slope of the linear relationship between the natural logarithm of SMR and the inverse of k·T [product of Boltzmann constant (k) and temperature (T) in Kelvin], respectively. We tested for differences between species’ SMR by including species as an interaction term in linear models.

Equation 5 \begin{equation*} SMR=a.{e}^{\raisebox{1ex}{$E$}\!\left/ \!\raisebox{-1ex}{$ kT$}\right.} \end{equation*}

Differences in MMR and AS data between species were tested by modelling a second-order polynomial relationship between metabolic data and temperature, including species as an interaction term, and a variance structure weighted by temperature and species. A generalized least squares (GLS) modelling approach was implemented using the nlme package (Pinheiro *et al.,* 2017) to account for data heteroscedasticity in R version 4.2.1 (R Core Team, 2022). Orthogonal polynomials were used for statistical inference to reduce the effect of collinearity among explanatory polynomial terms (Rawlings *et al.,* 1998). Due to the low number of hybrids in the sample, it was not possible to include these in the model.

## Results

### Genetic results

Amplification of a fragment of 236 base pairs (bp) of the mtDNA COI revealed 18 haplotypes, with two clear groups emerging, corresponding with previous morphological identification, because all *A. coronus* and *A. inodorus* individuals clustered within their respective species (Supplementary Fig. S3).

STRUCTURE analyses revealed two groups corresponding to morphologically identified *A. coronus* and *A. inodorus* (Supplementary Fig. S4). Nevertheless, assignment of individuals to species and identification of putative hybrids using STRUCTURE and NewHybrids consistently pointed to the presence of three *A. inodorus* individuals (of 57) with admixed origins (Table 1, Supplementary Table S5). For *A. coronus*, two individuals (of 17) were consistently identified as second-generation hybrids in NewHybrids and STRUCTURE (Table 1). Simulation studies showed that power to detect hybrids decreased with increased admixture (100% F1 and 98% for F2, for both STRUCTURE and NewHybrids, 90% for backcrosses with *A. coronus* and 86% for backcrosses with *A. inodorus* for NewHybrids, but 10% for backcrosses with *A. coronus* and 50% for backcrosses with *A. inodorus* for STRUCTURE).

**Table 1:** Summary of multiple lines of evidence for hybridization between *Argyrosomus coronus* and *A. inodorus*, including morphology, mtDNA COI sequence and nuclear microsatellite genotypic assignment using STRUCTURE (for model AD + IAF = admixture with independent allele frequencies) and NewHybrids

Individual	Morphology	mtDNA	STRUCTURE (q) (AD + IAF)	NewHybrids
T2C1	*A. inodorus*	*A. inodorus*	0.844	F2
T3C2	*A. inodorus*	*A. inodorus*	0.834	F2
T9C3	*A. inodorus*	*A. inodorus*	0.864	F2
T2C2	*A. coronus*	*A. coronus*	0.668	F2
T5C3	*A. coronus*	*A. coronus*	0.721	F2

### Experimental fish

In total, 74 individuals were captured, of which 57 were genetically identified as *A. inodorus* and 17 as *A. coronus.* Here, three *A. inodorus* individuals and two *A. coronus* individuals seemed consistently as F2 hybrids. *Argyrosomus inodorus* individuals ranged in TL from 38.9 to 72.5 cm (mean = 51 cm; SD = 7 cm) with fish mass ranging from 454 to 2875 g (mean = 1130.5 g; SD = 496 g). *Argyrosomus coronus* individuals ranged in TL from 31.5 to 71.7 cm (mean = 53 cm; SD = 11 cm) with mass ranging from 291 to 2878 g (mean = 1589.4 g; SD = 934.9 g). There was no significant difference in TL between *A. coronus* and *A. inodorus* (two-tailed *t-*test, *P* > 0.05 = 0.52), and no significant difference in mass between the two species (two-tailed *t-*test, *P* > 0.05 = 0.08).

### Metabolic rates

For the analysis, 39 *A. inodorus*, 14 *A. coronus* and 3 hybrids were used. The mass scaling exponent for SMR was estimated as 0.71 (SE = 0.26) for *A. coronus*, 1.05 (SE = 0.32) for *A. inodorus* and 0.91 for the hybrids. The mass scaling exponent for MMR was estimated as 0.83 (SE = 0.18) for *A. coronus*, 0.82 (SE = 0.27) for *A. inodorus* and 0.62 for the hybrids.

**Figure 2:**
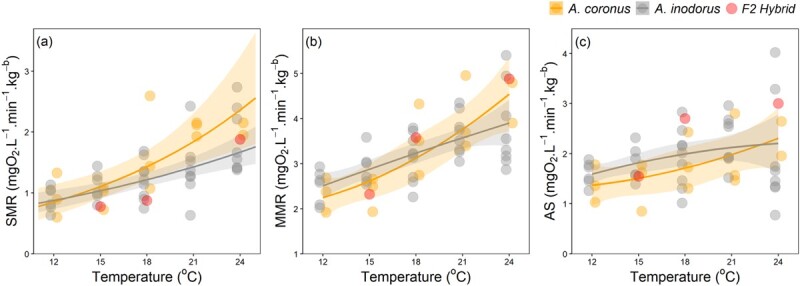
Physiological rates: (a) SMR (mgO_2_·L^−1^·min^−1^·kg^-b^), (b) MMR (mgO_2_·L^−1^·min^−1^·kg^-b^) and (c) AS (mgO_2_·L^−1^·min^−1^·kg^-b^) modelled against temperature (°C). Individual data (solid points) for *Argyrosomus coronus* (orange) and *A. inodorus* (silver) and their modelled (SMR: linear function of Arrhenius; MMR: GLS quadratic polynomial) relationship with temperature (solid lines) with 95% confidence intervals shaded. F2 hybrids are represented by the red points.

Standard and maximum metabolic rates increased with temperature (Supplementary Fig. S2). The rate of change for SMR increased with temperature for *A. coronus, A. inodorus* and the hybrids. The rate of change in MMR increased with temperature for *A. coronus* but remained relatively constant for *A. inodorus* and the hybrids. There was no significant effect of species (*A. coronus/A. inodorus*) on the relationship between SMR and temperature (Arrhenius: *P* = 0.139, Table 2) or between MMR and temperature (GLS: *P* = 0.871, Table 3). The modelled AS of *A. inodorus* was notably higher at cooler temperatures (12, 15, 18 and 21°C) compared with that of *A. coronus*, whereas the AS was similar at 24°C (Fig. 2). Despite these differences, species (*A. coronus/A. inodorus*) did not have a significant effect on the relationship between AS and temperature (GLS: *P* = 0.155, Table 4). The SMR of the hybrids fell below the 95% confidence intervals of the models for both species at 15 and 18°C, whereas the MMR fell within the upper 95% confidence interval of the models for both species at 18 and 24°C (Fig. 2). The AS of the hybrids also fell above the 95% confidence intervals of the models for both species at 18 and 24°C, whereas the individual at 15°C was in the upper bound of the 95% confidence interval (Fig. 2).

**Table 2:** Modelling results of natural logarithm of SMR (mgO_2_·L^−1^·min^−1^·kg^-b^) of *Argyrosomus coronus* and *A. inodorus* as a linear function of Arrhenius temperature (inverse of temperature in Kelvin multiplied by Boltzmann constant) with *A. coronus/A. inodorus* (species) as an interaction term, SE = standard error, and significant *P*-values are highlighted in bold. R2 = 0.44, degrees of freedom = 3, 49, F-statistic = 14.62

Effect	Estimate	SE	*t*-value	*P*-value
Intercept	24.960	5.412	4.612	**0.000**
Species	−9.516	6.322	−1.505	0.139
Arrhenius Temp	−0.617	0.136	−4.552	**0.000**
Species: Arrhenius Temp	0.234	0.159	1.479	0.146
Residual SE	0.283			

**Table 3:** GLS modelling results for MMR (mgO_2_·L^−1^·min^−1^·kg^-b^) of *Argyrosomus coronus* and *A. inodorus* presented as a quadratic function of temperature (Temp), with *A. coronus/A. inodorus* (species) as an interaction term, SE = standard error, and significant *P*-values are highlighted in bold

Effect	Estimate	SE	*t*-value	*P*-value
Intercept	3.314	0.174	19.076	**0.000**
Species	−0.031	0.191	−0.164	0.871
Temp	5.671	1.119	5.067	**0.000**
Temp2	0.828	1.118	0.741	0.463
Species: temp	−2.115	1.296	−1.631	0.110
Species: temp2	−0.892	1.296	−0.688	0.495
AIC	106.450			
Residual SE	0.393			

**Table 4:** GLS modelling results for AS (mgO_2_·L^−1^·min^−1^·kg^-b^) of *Argyrosomus coronus* and *A. inodorus* presented as a quadratic function of temperature (Temp), with *A. coronus/A. inodorus* (species) as an interaction term, SE = standard error, and significant *P*-values are highlighted in bold

Effect	Estimate	SE	*t*-value	*P*-value
Intercept	1.784	0.134	13.271	**0.000**
Species	0.230	0.159	1.445	0.155
Temp	2.371	0.857	2.766	0.008
Temp2	0.537	0.899	0.598	0.553
Species: temp	−0.571	0.110	−0.514	0.610
Species: temp2	−0.766	1.112	−0.689	0.495
AIC	103.558			
Residual SE	0.392			

## Discussion

Although most physiology research to predict the response of species to climate change has focused on single species and population differences in physiological rates (Duncan *et al.,* 2019; Crear *et al.,* 2020), this study compared the AS between two congeners that overlap spatially, ecologically and form a common pool resource (Griffiths and Heemstra, 1995; Kirchner, 1999; Kirchner and Voges, 1999; Kirchner *et al.,* 2001; Potts *et al.,* 2010). This study found that the metabolic traits of these two species differ and explained both the distribution of the cool-temperate *A. inodorus* and the recent poleward dispersal of the warm-temperate *A. coronus* into the rapidly warming waters of central Namibia (Potts *et al.,* 2014a).

Although mechanistic models will be the next step for predicting the future distribution of these species, based on the findings of this study and the warming trend in northern Benguela, it is likely that the distribution of *A. inodorus* will contract southward, whereas *A. coronus* may extend its range further south and increase in abundance in central Namibia. However, both species may be restricted from a shift further southward by the cold water (<12°C) associated with the perennial Lüderitz Upwelling Cell (Fig. 1, Rhein *et al.,* 2013), which already acts as a barrier to the dispersal of a range of coastal species (Henriques *et al.,* 2014).

The Lüderitz Upwelling Cell is intensifying, with sea surface temperatures (SSTs) decreasing significantly between 1870 and 2006 (Narayan *et al.,* 2010). Besides presenting unfavourable conditions, further cooling in the Lüderitz Upwelling Cell may drive temperatures below the critical lower limits of these species. In a preliminary trial, two of the four *A. inodorus* lost equilibrium during SMR measurements, and none were able to recover from the exhaustive protocol to elicit MMR at 10°C. It is therefore likely that this feature may drive a northward shift of individuals along the southern edge of the distribution of both species. This is most concerning for *A. inodorus* because it will be subjected to a considerable coastal squeeze.

Adaptation may facilitate physiological changes that promote survival in the new environmental conditions, as shown in other species but typically occurs over longer timescales than needed to match the predicted environmental changes in this region (Munday, 2014). In the case of *A. inodorus,* its recent hybridization with *A. coronus* (Potts *et al.,* 2014b) may have provided a catalyst for adaptation. However, understanding whether hybridization and backcrossing may influence genes that affect the thermal tolerance and physiology of these species, or indeed for any fish species, is critical. Although preliminary, our findings showed that the F2 hybrids had notably high AS when compared with other individuals at 18 and 24°C. Grant and Grant (2010) and Hoffmann and Sgrό (2011) suggested that hybridization may be beneficial in the face of climate change because it may increase the evolutionary potential of populations to changing environments (*e.g.*Vedder *et al.,* 2022). Indeed, our initial findings support the suggestion and demonstrate that hybridization may be a powerful mechanism for *A. inodorus* and possibly other fishes to rapidly adapt their physiological phenotypes to the impacts of ocean warming. This may have major implications for our prediction of climate change impacts and research in ocean warming hotspots and should be prioritized for research to gain a better understanding of this potential mechanism.

Currently, there is little evidence to suggest that this hybridization event may result in the formation of a new species. When compared with the study by Potts *et al.* (2014b), who found that ~3% of the individuals sampled in the region were hybrids in 2009, the findings of this study suggest that the percentage of hybrids had increased to 7% by 2019. Although this was a single collection effort, these individuals were collected from a single locality through 4 months, providing a snapshot into the composition of these species.

Besides the development of a hybrid zone, the increasing overlap of the two *Argyrosomus* species in Namibia will also have consequences for resource competition. The diet of the two species is similar (Smale and Bruton, 1985; Potts *et al.,* 2010), and it is therefore likely that the species with the greatest energetic advantage will outcompete the other for the available prey. This study revealed that *A. inodorus* had a comparatively higher AS at cooler temperatures (<21°C), whereas the AS of *A. coronus* individuals at 24°C were on average higher. This suggests that *A. inodorus* may have a competitive advantage in much of central Namibia at present. However, as SSTs in this region increase beyond 21°C, *A. coronus* may outcompete *A. inodorus* for food resources.

The changing distributional patterns of the *Argyrosomus* species may have profound consequences for Namibia’s coastal fisheries. In Namibia, *A. inodorus* supports a large recreational shore-based and ski-boat fishery (Kirchner and Beyer, 1999), a small commercial boat-based hook and line fishery (Stage and Kirchner, 2005) and a small subsistence/artisanal fishery (Cardoso *et al.,* 2006). Presently, there are no conceptual objectives, operational objectives, performance indicators or reference points for the Namibian line fishery (Potts *et al.,* 2020; Marine Resources Act, 2000 (Act No. 27 of 2000)), and it is managed based on rules and regulations that were developed by scientists from the Namibian Ministry of Fisheries and Marine Resources in the 1990s. As the dominant coastal fishery species, the response of *A. inodorus* to the cooling in the south and warming in the north will largely dictate the future of Namibia’s coastal fisheries. Based on these findings, this species may occupy a smaller area, unless it is able to adapt. One behavioural adaptation may be to shift into deeper waters, as has been observed for other fishes (Perry *et al.,* 2005; Fredston-Hermann *et al.,* 2020). However, this is unlikely in Namibia primarily due to the anoxic zone that extends from 20 m (Griffiths, 1997). Although acclimation may allow *A. inodorus* to persist in this rapidly changing environment, it is ultimately bounded by the physiological phenotype (Munday, 2014), and it may not be sufficient to facilitate the long-term persistence of this species.

The southward distributional shift of *A. coronus* is likely to result in an increase in the abundance of these fishes in the WCRA and potentially improve fishing opportunities due to the large maximum size and fast growth of this species (Supplementary Table S1). As a result, this distributional shift may be advantageous for all the coastal fishery sectors (recreational, subsistence and commercial) in the short term. Although the increase in the abundance of the newly arriving *A. coronus* may benefit all sectors, the recreational fishery in particular may benefit due to an increase in trophy specimens. This may have positive consequences for the local economy due to the economic impact largely associated with foreign and local fishing tourism (Butler *et al.,* 2020; Kadagi *et al.,* 2020).

The concentration of *A. inodorus* and the influx of *A. coronus* into the WCRA will require careful management. The improved fishing opportunities are likely to increase recreational angling effort, whereas the coastal squeeze of *A. inodorus* into central Namibia will likely reduce the efficacy of marine protected areas and mining concessions that are closed to shore-based fishing and situated north and south of the contemporary *A. inodorus* distribution. These predicted changes in the distribution of these two species suggest that fisheries monitoring data should be interpreted with caution because changes in catch rate may not necessarily be related to changes in population abundance. This is particularly relevant because the recreational fishing effort is highest in summer, when water temperatures are high and could place *Argyrosomus* species under additional pressure post–catch-and-release. In addition, the traditional regulations associated with the *A. inodorus* fishery, such as size limits, may not be appropriate for the fishery as the proportion of *A. coronus* increases in the catch. Close monitoring of the catch composition (using genetic techniques) in the WCRA will be necessary to ensure that proactive amendments to the regulations are made to adequately protect this new fisheries resource in Namibia.

## Funding

This work was supported by the National Research Foundation—National Commission on Research Science and Technology, South Africa—Namibia Agreement on Cooperation in Science and Technology [reference number NAMG160519165537, grant number 105945].

## Author Contributions

B.A.P.: Conceptualization, methodology, investigation, data curation, formal analysis, writing—original draft. M.I.D.: Conceptualization, methodology, investigation, data curation, formal analysis, writing—review & editing. A.C.W.: Conceptualization, methodology, investigation, writing—review & editing. S.M.: Conceptualization, writing—review & editing. C.J.: Methodology, investigation—review & editing. N.J.M.K.: Methodology, investigation, data curation, formal analysis, writing—review & editing. P.W.S.: Methodology, investigation, data curation, formal analysis, writing—review & editing. R.H.: Methodology, investigation, data curation, formal analysis, writing—review & editing. W.M.P.: Conceptualization, methodology, investigation, data curation, formal analysis, writing—review & editing.

## Data Availability

The data underlying this article are available in the article and in its online supplementary material.

## Supplementary Material

Web_Material_coad026
