# Correction to: Ocean warming favours a northern *Argyrosomus* species over its southern congener, whereas preliminary metabolic evidence suggests that hybridization may promote their adaptation

**DOI:** 10.1093/conphys/coad050

**Published:** 2023-07-03

*Conserv Physiol* 11(1): coad026; doi:10.1093/conphys/coad026.

In the originally published version of this manuscript, in the ‘Cite as’ section, there was a typographical error in the name of author Brett A. Pringle.

The section has been corrected to read as follows: Cite as: Pringle BA, Duncan MI, Winkler AC, Mafwila S, Jagger C, McKeown NJ, Shaw PW, Henriques R, Potts WM (2023) Ocean warming favours a northern *Argyrosomus* species over its southern congener, whereas preliminary metabolic evidence suggests that hybridization may promote their adaptation. *Conserv Physiol* 11(1): coad026; doi:10.1093/conphys/coad026.

