# Supplementary material for: Ocean warming favours a northern Argyrosomus species over its southern congener, whereas preliminary metabolic evidence suggests that hybridization may promote their adaptation
Source: Conserv Physiol. 2023 Jul 3;11(1):coad026. doi: 10.1093/conphys/coad026 (PMC10170327; doi:10.1093/conphys/coad026)
Supplement: Web_Material_coad026 [file web_material_coad026.pdf]

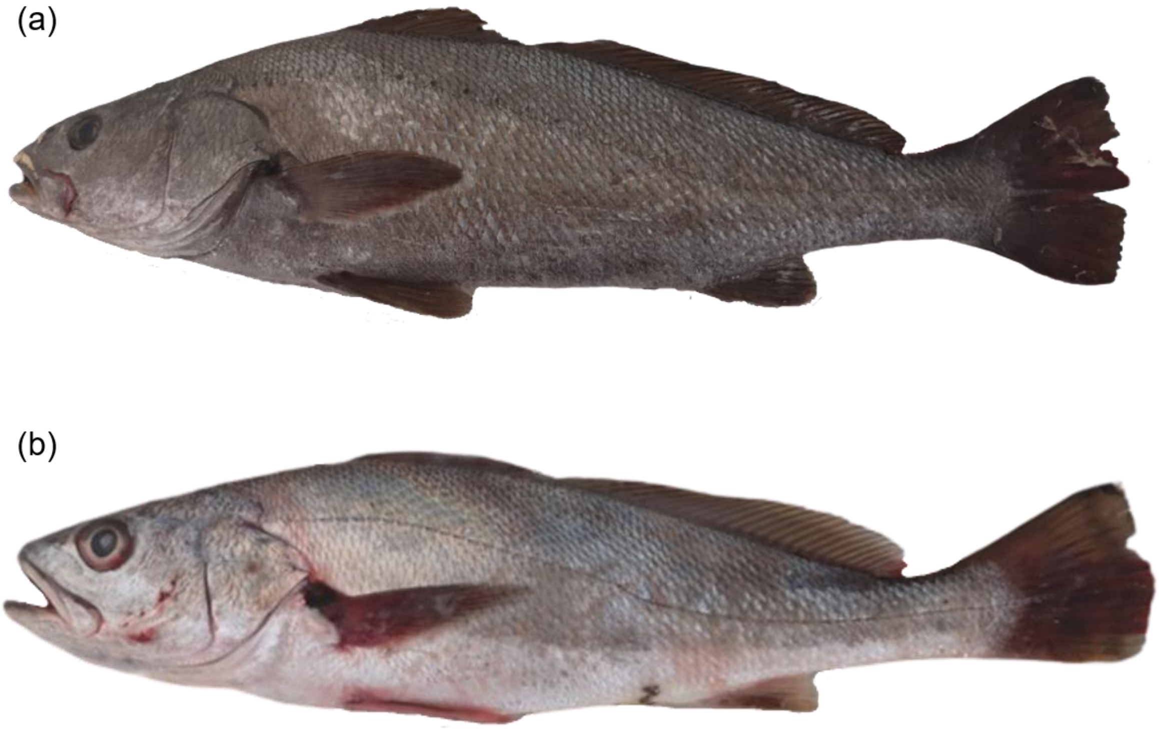

Figure S1: Photographs of (a) *Argyrosomus coronus*, 63 cm total length (TL) and (b) *A. inodorus*, 42 cm Total Length (TL), caught at the Toscanini old diamond mine in the Skeleton Coast National Park (SCNP), Namibia.

Table S1: Life-history characteristics of *Argyrosomus coronus* and *A. inodorus* (Adapted from Potts *et al.,* 2010). TL = Total Length, L_{50} = Length at 50% maturity; A_{50} = Age at 50% maturity; M = Male; F = Female.

Species	Country	Max. TL (mm)	Max. weight (kg)	Max. age (y)	L_{50} (mm)	A_{50} (y)	Sex ratio M:F
A. coronus	Angola	1900[1]	77[1]	13[5]	870[5]	4–5[5]	1:1.4[5]
A. inodorus	Namibia	1200[2]	36[1]	28[3]	350-360[4]	1.5–1.6[4]	-

[1]Griffiths and Heemstra, 1995; [2]Kirchner, 1999; [3]Kirchner and Voges, 1999; [4]Kirchner *et al.,* 2001; [5]Potts *et al.,* 2010

Table S2: Percentage of *Argyrosomus coronus* (numbers sampled in parentheses) in the catch composition of the *Argyrosomus* fishery in the West Coast Recreational Area (WCRA) and Skeleton Coast National Park (SCNP) in Namibia and in the Cunene River mouth (border between Angola and Namibia) (Extracted from Potts *et al.,* 2014b).

	Namibia		Namibia-Angola
Period	WCRA	SCNP	Cunene River Mouth
1993-1995	8 (237*)	15 (325*)	99 (258*)
2008-2009	57 (146† + 18‡)	-	100 (12‡)

*Allozyme electrophoresis-validated otolith morphometry.
†Molecular Polymerase Chain Reaction-based Restriction Fragment Length Polymorphism of mtDNA.
‡MtDNA sequencing.

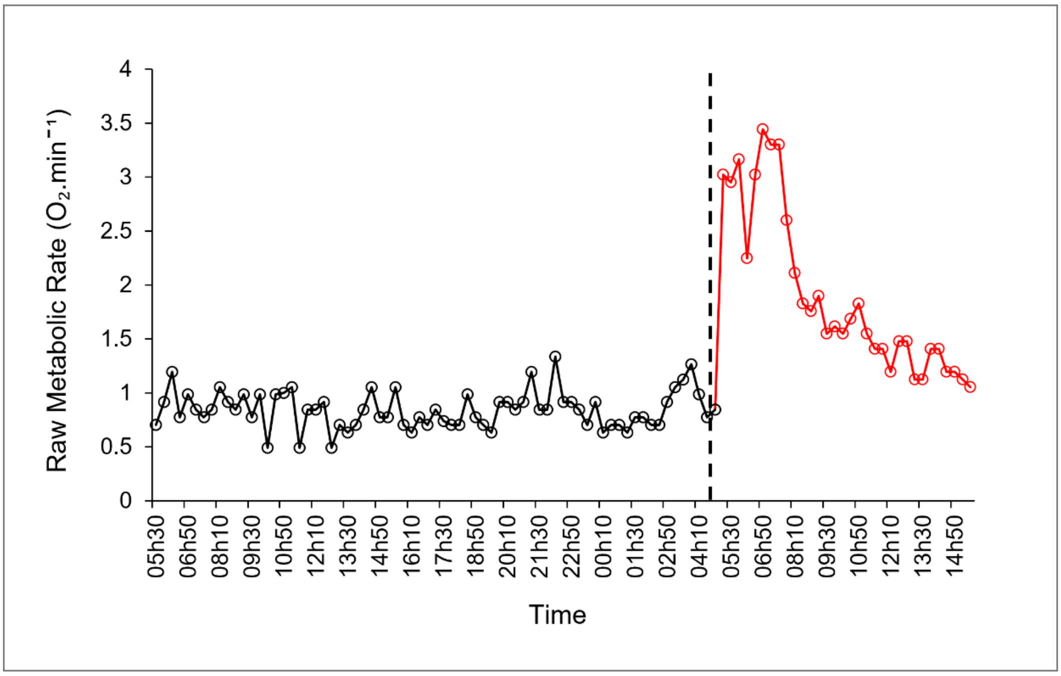

Figure S2: An example of a full metabolic rate trial at 18 ˚C for *Argyrosomus inodorus*. Each black point corresponds to a calculated raw metabolic rate (RO_2) before chasing (black dashed line). Each red point corresponds to a calculated raw metabolic rate (MO_2) post exhaustive protocol and the subsequent recovery.

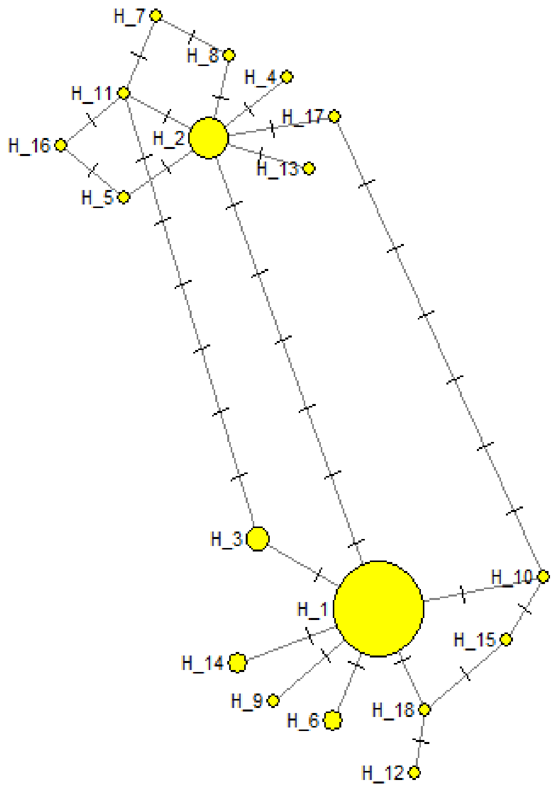

Figure S3: Haplotype network for two *Argyrosomus* species sampled in this study. *Argyrosomus inodorus* clade; Haplotypes 1, 3, 6, 9, 10, 12, 14, 15, 18 and *Argyrosomus coronus* clade; Haplotypes 2, 4, 5, 7, 8, 11,13, 16, 17.

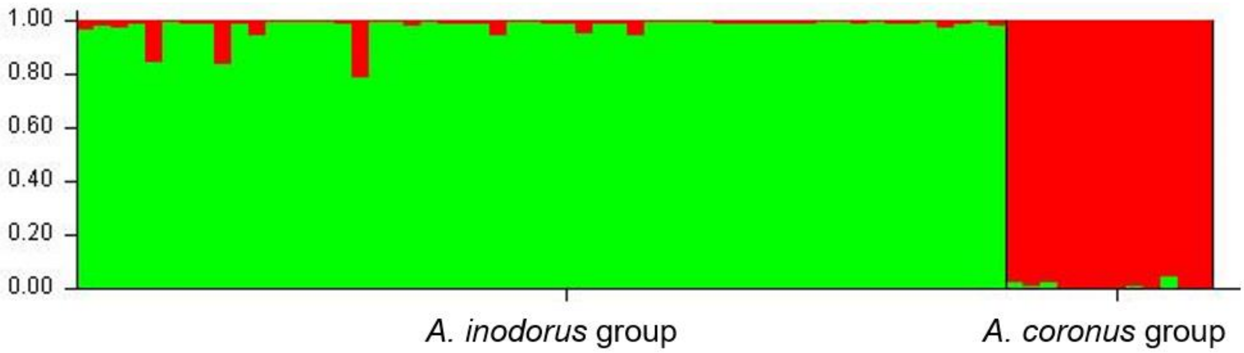

Figure S4: Multilocus genetic tests performed on 74 *Argyrosomus* species individuals sampled in the study, using STRUCTURE. Green vertical bars indicate *Argyrosomus inodorus* type individuals while the red bars indicate *Argyrosomus coronus* types.

Nucleotide (π) and haplotype (h) diversity were higher for *A. coronus* in comparison with *A. inodorus* (π = 0.0040, h = 0.730; vs π = 0.0018, h = 0.370, respectively). Summary indices of variability for the *A. inodorus* and *A. coronus* groups are reported in Table S2. The multilocus F_{ST} between both groups was 0.24. Microsatellite-derived genotype proportions conformed to Hardy-Weinberg equilibrium for each species group vs. locus combination, with the exception of UBA853 for the *A. inodorus* group (P = 0.018).

Table S3: Genetic variability for the resolved *Argyrosomus inodorus* and *Argyrosomus coronus* groups as summarised using allele number (N_A), allelic richness (A_R) and observed and expected heterozygosity (H_O and H_E, respectively), based on five microsatellite loci

	Species	
Average across loci	*Argyrosomus inodorus*	*Argyrosomus coronus*
N	57	17
N_A	11	6.8
A_R	7.9	6.8
H_O	0.63	0.66
H_E	0.69	0.72

Table S4: Assignment results for the simulated dataset obtained with HybridLab, for four hybrid categories: F1 (*A. coronus* vs *A. inodorus*), F2 (F1 vs F1), backcross *A. coronus* (F2 vs *A. coronus*) and backcross *A. inodorus* (F2 vs *A. inodorus*), based on five microsatellite loci. *with probability of assignment to the right category >75%, majority had a higher probability of being a hybrid of their own category than any other category. bc = backcross.

		Percent of correctly identified individuals (parental species or hybrids) (%)					
		A. coronus	*A. inodorus*	F1	F2	bc *A. coronus*	bc *A. inodorus*
F1	STRUCTURE	97	94	100	x	x	x
	NewHybrids	100	100	100	x	x	x
F2	STRUCTURE	95	96	x	98	x	x
	NewHybrids	80	91	x	98	x	x
backcross *A. coronus*	STRUCTURE	100	100	x	x	10	x
	NewHybrids	66	100	x	x	90	x
backcross *A. inodorus*	STRUCTURE	100	100	x	x	x	50
	NewHybrids	100	59	x	x	x	86

Table S4: Summary of multiple lines of evidence for hybridization between *Argyrosomus coronus* and *Argyrosomus inodorus*, including morphology, mtDNA COI haplotypes and nuclear microsatellite genotypic assignment using STRUCTURE (for models AD+IAF = admixture with independent allele frequencies; NAD+IAF = no admixture with independent allele frequencies; AD+CAF = admixture with correlated allele frequencies) and NewHybrids**.** Bold = F2 hybrid. bc = backcross.

	**Structure (q)**						**NewHybrids**					
	AD+IAF		NAD+IAF		AD+CAF							
Individual	*A. inodorus*	*A. coronus*	*A. inodorus*	*A. coronus*	*A. inodorus*	*A. coronus*	*A. coronus*	*A. inodorus*	F1	F2	bc_*A. coronus*	bc_*A. inodorus*
**T2C2**	**0.332**	**0.668**	**0.133**	**0.867**	**0.321**	**0.679**	**0.28794**	**0.02613**	**0.00096**	**0.64089**	**0.03689**	**0.00719**
T3C3	0.008	0.992	0	1	0.008	0.992	0.98662	0	0	0.0082	0.00517	0
T4C3	0.016	0.984	0	1	0.013	0.987	0.98348	0	0.00005	0.01063	0.00583	0.00001
**T5C3**	**0.279**	**0.721**	**0**	**1**	**0.255**	**0.745**	**0.08043**	**0.00001**	**0.00023**	**0.80983**	**0.10924**	**0.00026**
T7C4	0.01	0.99	0	1	0.01	0.99	0.98501	0	0.0001	0.01058	0.00425	0.00007
T12C2	0.007	0.993	0	1	0.007	0.993	0.99572	0	0	0.00246	0.00182	0
T13C1	0.005	0.995	0	1	0.005	0.995	0.99866	0	0	0.00052	0.00081	0
T14C1	0.076	0.924	0	1	0.073	0.927	0.78446	0	0.00001	0.20988	0.00555	0.0001
T14C2	0.005	0.995	0	1	0.005	0.995	0.99882	0	0	0.00044	0.00073	0
T15C3	0.005	0.995	0	1	0.005	0.995	0.99895	0	0	0.00035	0.00071	0
T16C2	0.005	0.995	0	1	0.005	0.995	0.9981	0	0	0.00084	0.00106	0
T16C3	0.005	0.995	0	1	0.005	0.995	0.9987	0	0	0.00048	0.00082	0
T18C2	0.006	0.994	0	1	0.005	0.995	0.99707	0	0	0.00142	0.00151	0
T18C3	0.005	0.995	0	1	0.004	0.996	0.99844	0	0	0.00066	0.0009	0
T19C2	0.022	0.978	0	1	0.018	0.982	0.95394	0.00001	0.0003	0.03574	0.0098	0.00021
T19C3	0.004	0.996	0	1	0.004	0.996	0.99896	0	0	0.00038	0.00066	0
T20C2	0.005	0.995	0	1	0.005	0.995	0.99889	0	0	0.00041	0.0007	0
T1C1	0.974	0.026	1	0	0.974	0.026	0	0.9213	0.00003	0.04286	0.00001	0.03581
T1C2	0.986	0.014	1	0	0.987	0.013	0	0.9686	0	0.00828	0	0.02311
T1C3	0.98	0.02	1	0	0.98	0.02	0	0.95169	0	0.02273	0	0.02557
T1C4	0.992	0.008	1	0	0.992	0.008	0	0.98448	0	0.00612	0	0.00939
**T2C1**	**0.844**	**0.156**	**1**	**0**	**0.845**	**0.155**	**0**	**0.2942**	**0**	**0.56036**	**0**	**0.14543**
T2C3	0.995	0.005	1	0	0.995	0.005	0	0.99768	0	0.00058	0	0.00174
T2C4	0.994	0.006	1	0	0.994	0.006	0	0.99582	0	0.00128	0	0.0029
T3C1	0.995	0.005	1	0	0.995	0.005	0	0.99652	0	0.00122	0	0.00226
**T3C2**	**0.834**	**0.166**	**1**	**0**	**0.844**	**0.156**	**0**	**0.42362**	**0**	**0.56564**	**0.00002**	**0.01073**
T3C4	0.984	0.016	1	0	0.985	0.015	0	0.97844	0	0.01368	0	0.00788
T4C1	0.961	0.039	1	0	0.963	0.037	0	0.74469	0.0001	0.17426	0.00002	0.08093
T5C1	0.995	0.005	1	0	0.995	0.005	0	0.99797	0	0.00052	0	0.00151
T5C2	0.995	0.005	1	0	0.995	0.005	0	0.99753	0	0.00073	0	0.00174

T5C4	0.995	0.005	1	0	0.996	0.004	0	0.99804	0	0.00043	0	0.00153
T6C1	0.996	0.004	1	0	0.996	0.004	0	0.99877	0	0.00025	0	0.00098
T6C2	0.994	0.006	1	0	0.994	0.006	0	0.92888	0	0.02908	0	0.04204
T6C3	0.94	0.06	1	0	0.941	0.059	0	0.02557	0	0.96607	0.00002	0.00833
T6C4	0.993	0.007	1	0	0.994	0.006	0	0.99618	0	0.00121	0	0.00261
T7C2	0.993	0.007	1	0	0.992	0.008	0	0.98899	0	0.00604	0	0.00497
T7C3	0.993	0.007	1	0	0.993	0.007	0	0.98333	0.00001	0.00996	0.00001	0.00669
T8C2	0.995	0.005	1	0	0.994	0.006	0	0.99422	0	0.00195	0	0.00383
T8C3	0.992	0.008	1	0	0.993	0.007	0	0.997	0	0.00098	0	0.00202
T8C4	0.992	0.008	1	0	0.992	0.008	0	0.99577	0	0.00158	0	0.00265
T9C2	0.981	0.019	1	0	0.981	0.019	0	0.97174	0	0.01498	0	0.01328
**T9C3**	**0.864**	**0.136**	**0.998**	**0.002**	**0.866**	**0.134**	**0**	**0.44223**	**0.00013**	**0.50009**	**0.00026**	**0.05729**
T9C4	0.994	0.006	1	0	0.994	0.006	0	0.99644	0	0.00113	0	0.00243
T10C2	0.995	0.005	1	0	0.995	0.005	0	0.99634	0	0.00118	0	0.00247
T10C3	0.994	0.006	1	0	0.994	0.006	0	0.9938	0	0.00228	0	0.00392
T10C4	0.992	0.008	1	0	0.993	0.007	0	0.98135	0	0.00978	0	0.00887
T11C1	0.977	0.023	1	0	0.979	0.021	0	0.78554	0.00001	0.13149	0	0.08295
T11C2	0.994	0.006	1	0	0.994	0.006	0	0.99417	0	0.00232	0	0.00351
T11C3	0.994	0.006	1	0	0.994	0.006	0	0.99749	0	0.00069	0	0.00182
T11C4	0.98	0.02	1	0	0.981	0.019	0	0.79153	0.00005	0.11978	0.00001	0.08863
T12C3	0.996	0.004	1	0	0.996	0.004	0	0.99875	0	0.00024	0	0.00101
T12C4	0.994	0.006	1	0	0.994	0.006	0	0.99567	0	0.00146	0	0.00287
T13C2	0.996	0.004	1	0	0.996	0.004	0	0.999	0	0.00017	0	0.00083
T13C4	0.995	0.005	1	0	0.995	0.005	0	0.99861	0	0.0003	0	0.00109
T14C3	0.987	0.013	1	0	0.988	0.012	0	0.93826	0.00001	0.0295	0	0.03223
T14C4	0.993	0.007	1	0	0.993	0.007	0	0.99438	0	0.00192	0	0.0037
T15C1	0.995	0.005	1	0	0.995	0.005	0	0.99831	0	0.00055	0	0.00114
T15C2	0.991	0.009	1	0	0.991	0.009	0	0.98039	0	0.00961	0	0.00999
T15C4	0.988	0.012	1	0	0.988	0.012	0	0.99008	0	0.00363	0	0.00629
T16C1	0.969	0.031	1	0	0.968	0.032	0	0.99638	0	0.00122	0	0.0024
T16C4	0.994	0.006	1	0	0.995	0.005	0	0.99769	0	0.00079	0	0.00152
T17C2	0.995	0.005	1	0	0.995	0.005	0	0.99789	0	0.00048	0	0.00162
T17C3	0.994	0.006	1	0	0.994	0.006	0	0.99336	0	0.00258	0	0.00406
T17C4	0.995	0.005	1	0	0.995	0.005	0	0.99792	0	0.00047	0	0.0016

T18C1	0.994	0.006	1	0	0.994	0.006	0	0.99112	0.00002	0.00413	0	0.00474
T18C4	0.995	0.005	1	0	0.995	0.005	0	0.98457	0	0.00638	0	0.00905
T19C1	0.993	0.007	1	0	0.993	0.007	0	0.99188	0	0.00345	0	0.00467
T19C4	0.983	0.017	1	0	0.985	0.015	0	0.97096	0	0.00729	0	0.02175
T20 C1	0.995	0.005	1	0	0.995	0.005	0	0.9947	0	0.0016	0	0.0037
T20 C3	0.995	0.005	1	0	0.995	0.005	0	0.99704	0	0.00089	0	0.00207
T20 C4	0.981	0.019	1	0	0.981	0.019	0	0.98793	0	0.00733	0	0.00474
t21c2	0.995	0.005	1	0	0.995	0.005	0	0.99695	0	0.00088	0	0.00217
t21c3	0.993	0.007	1	0	0.993	0.007	0	0.99421	0	0.0015	0	0.0043
t21c4	0.995	0.005	1	0	0.996	0.004	0	0.9988	0	0.00023	0	0.00097

TABLE S6. Checklist of 53 essential criteria for the reporting of methods for aquatic intermittent-flow respirometry.

Number	Criterion and Category	Response
	**EQUIPMENT, MATERIALS, AND SETUP**	
**1**	Body mass of animals at time of respirometry	Measured immediately after respirometry
**2**	Volume of empty respirometers	Small: 7.62 L<br>Medium: 21.26 L<br>Large: 44.65 L
**3**	How chamber mixing was achieved	Internally connected pumps (small: 600L/h 12w 1m AC SOBO water pump, medium: 880L/h 15w 1.2m AC SOBO water pump, large: 1200L/h 25w 1.5m AC SOBO water pump) mixed water within respirometers to minimise water stratification
**4**	Ratio of net respirometer volume (plus any associated tubing in mixing circuit) to animal body mass	Small 16.48:1<br>Medium 20.17:1<br>Medium 19.10:1<br>Large 23.06:1
**5**	Material of tubing used in any mixing circuit	Oxygen-impermeable clear thick-wall hose
**6**	Volume of tubing in any mixing circuit	Mean: 0.65 L<br>SD: 0.09 L
**7**	Confirm volume of tubing in any mixing circuit was included in calculations of oxygen uptake	Confirmed
**8**	Material of respirometer (e.g. glass, acrylic, etc.)	Thick-wall Perspex
**9**	Type of oxygen probe and data recording	Optical oxygen sensor (OXFTC, Pyro Science GmbH). FireStingO2 fibre optic oxygen meter (FSO2-4, Pyro Science GmbH) with bare optical fibres (SPFIB-BARE, Pyro Science GmbH. Recorded using Pyro Oxygen Logger Software (Pyro Science GmbH)
**10**	Sampling frequency of water dissolved oxygen	5 seconds
**11**	Describe placement of oxygen probe (in mixing circuit or directly in chamber)	In mixing circuit, where the peristaltic pump drew water from and fed through flow-through cells with an optical oxygen sensor
**12**	Flow rate during flushing and recirculation, or confirm that chamber returned to normoxia during flushing	Chamber returned to normoxia during flushing
**13**	Timing of flush/closed cycles	15-minute flush (on) and 5-minute closed/measurement (off) cycle
**14**	Wait (delay) time excluded from closed measurement cycles	1 minute
**15**	Frequency and method of probe calibration (for both 0 and 100% calibrations)	Before each trial. Point calibration in saturated water
**16**	State whether software temperature compensation was used during recording of water oxygen concentration	Used the FireStingO2 temperature probe and software to adjust O_2 measurements. No other temperature compensation
	**MEASUREMENT CONDITIONS**	
**17**	Temperature during respirometry	12, 15, 18, 21 or 24˚C depending on the treatment.
**18**	How temperature was controlled	Using a heat pump (AQUAHEAT SF020P)

**19**	Photoperiod during respirometry	Under natural light conditions (sunroof)
**20**	If (and how) ambient water bath was cleaned and aerated during measurement of oxygen uptake (e.g. filtration, periodic or continuous water changes)	Complete replacement of water within the experimental system (water bath and chambers) and use of chlorine to clean tanks was conducted prior to each trial
**21**	Total volume of ambient water bath and any associated reservoirs	2240 L
**22**	Minimum water oxygen dissolved oxygen reached during closed phases	3.5 mg/l at 24 ˚C (41% saturation). In general, measurements never went below 80% saturation unless the animal was stressed (typically at the beginning of the trial or during MMR protocol)
**23**	State whether chambers were visually shielded from external disturbance	Confirmed
**24**	How many animals were measured during a given respirometry trial (i.e. how many animals were in the same water bath)	4 (2 per bath) in a connected system
**25**	If multiple animals were measured simultaneously, state whether they were able to see each other during measurements	Not able to see each other
**26**	Duration of animal fasting before placement in respirometer	36 hours
**27**	Duration of all trials combined (number of days to measure all animals in the study)	15/04/2019-02/08/2019
**28**	Acclimation time to the laboratory (or time since capture for field studies) before respirometry measurements	Wild captured<br>Mean: 48.5 days<br>Range: 28-76 days
	**BACKGROUND RESPIRATION**	
**29**	State whether background microbial respiration was measured and accounted for, and if so, method used (e.g. parallel measures with empty respirometry chamber, measurements before and after for all chambers while empty, both)	Measured and accounted for. Measurements after for all chambers while empty
**30**	State if background respiration was measured at beginning and/or end, state how many slopes and for what duration	End of trial<br>3-hour blank with closed chambers (one continuous slope)
**31**	State how changes in background respiration were modelled over time (e.g. linear, exponential, parallel measures)	Parallel<br>We used a single blank at the end of the trial and assumed that represented background respiration throughout the trial
**32**	Level of background respiration (e.g. as a percentage of SMR)	<1%
**33**	Method and frequency of system cleaning (e.g. system bleached between each trial, UV lamp)	The system was completely flushed, bleached and replaced with fresh seawater between each trial
	**STANDARD OR ROUTINE METABOLIC RATE**	
**34**	Acclimation time after transfer to chamber, or alternatively, time to reach beginning of metabolic rate measurements after introduction to chamber	12 hours
**35**	Time period, within a trial, over which oxygen uptake was measured (e.g. number of hours)	22 hours

**36**	Value taken as SMR/RMR (e.g. quantile, mean of lowest 10 percent, mean of all values)	The SMR was estimated as the quantile assigned to the bottom 20 % of the RO_2 data, as the coefficient of variation between readings was above the suggested 5.4 % threshold, following the guidelines of Chabot et al. (2016)
**37**	Total number of slopes measured and used to derive metabolic rate (e.g. how much data were used to calculate quantiles)	We required a minimum of 25 measurements above our R^2 threshold to estimate SMR. The majority of fish (45/56) had more than 50 slopes<br>Minimum threshold: >25<br>Range: 26-73
**38**	Whether any time periods were removed from calculations of SMR/RMR (e.g. data during acclimation, periods of high activity [e.g. daytime])	N/A
**39**	r^2 threshold for slopes used for SMR/RMR (or mean)	0.9
**40**	Proportion of data removed due to being outliers below r-squared threshold	We tested 63 healthy specimens, 56 produced > 25 SMR measurements above the r^2 threshold and were included in the analysis. Of those 56 fish, MR measurement exclusion ranged from 0% to 64% with the majority of trials (45/56) excluding no more than 7% of the data
	**MAXIMUM METABOLIC RATE**	
**41**	When MMR was measured in relation to SMR (i.e. before or after)	After
**42**	Method used (e.g. critical swimming speed respirometry, swim to exhaustion in swim tunnel, or chase to exhaustion)	Chasing and tail grabbing (3 minutes) and exposed to air (1 minute)
**43**	Value taken as MMR (e.g. the highest rate of oxygen uptake value after transfer, average of highest values)	Estimated from the single steepest decline in oxygen concentration during ten hours of intermittent-flow respirometry after the chasing protocol
**44**	If MMR measured post-exhaustion, length of activity challenge or chase (e.g. 2 min, until exhaustion, etc.)	3 minutes of chasing and tail grabbing
**45**	If MMR measured post-exhaustion, state whether further air-exposure was added after exercise	1 minute of air exposure
**46**	If MMR measured post-exhaustion, time until transfer to chamber after exhaustion or time to start of oxygen uptake recording	<20 seconds
**47**	Duration of slopes used to calculate MMR (e.g. 1 min, 5 min, etc.)	5 min
**48**	Slope estimation method for MMR (e.g. rolling regression, sequential discrete time frames)	Linear regression
**49**	How absolute aerobic scope and/or factorial aerobic scope is calculated (i.e. using raw SMR and MMR, allometrically mass-adjusted SMR and MMR, or allometrically mass-adjusting aerobic scope itself)	Allometrically mass-adjusted SMR and MMR
	**DATA HANDLING AND STATISTICS**	
**50**	Sample size	n = 56<br>12 ˚C (*A. inodorus* n = 6; *A. coronus* n = 3)<br>15 ˚C (*A. inodorus* n = 6; *A. coronus* n = 3; hybrids n = 1)<br>18 ˚C (*A. inodorus* n = 9; *A. coronus* n = 3; hybrids n = 1)<br>21 ˚C (*A. inodorus* n = 8; *A. coronus* n = 3)<br>24 ˚C (*A. inodorus* n = 8; *A. coronus* n = 2; hybrids n = 1)

**51**	How oxygen uptake rates were calculated (software or script, equation, units, etc.)	Equation
**52**	Confirm that volume (mass) of animal was subtracted from respirometer volume when calculating oxygen uptake rates	Confirmed
**53**	State whether analyses accounted for variation in body mass and describe any allometric mass-corrections or adjustments	To estimate the mass-scaling exponent for the metabolic rate of *Argyrosomus* species, the data were first temperature-corrected by dividing metabolic rate data by the Arrhenius function. The slope of the linear regression between the natural logarithm of RO_2 (temperature corrected) and the natural logarithm of mass was taken as the allometric exponent (α) of the mass scaling relationship for either SMR or MMR data. RO_2 data was then mass corrected (MO_2) using the mass scaling relationship

Supplementary data

species	temp	smr	mmr	as	temp0	codetreat
coronus	12	1.26299637369555	2.68593802780477	1.42294165410923	0	c12
coronus	12	0.60633039701856	2.37969204022506	1.7733616432065	0	c12
coronus	12	0.942170630485025	1.91943005983636	0.977259429351334	0	c12
coronus	15	1.07143961244114	1.9325444159286	0.86110480348746	3	c15
coronus	15	0.733994808656374	2.50043386788513	1.76643905922876	3	c15
coronus	15	1.05353986172289	2.65716483546248	1.60362497373959	3	c15
coronus	18	2.63829190717025	4.3255210148969	1.68722910772664	6	c18
coronus	18	1.08594007197148	3.50529209588131	2.41935202390982	6	c18
coronus	18	1.50050725104041	2.74470723010197	1.24419997906155	6	c18
coronus	21	2.21720156617231	4.95472680560543	2.73752523943312	9	c21
coronus	21	2.1438280288813	3.68297660769075	1.53914857880945	9	c21
coronus	21	1.95640390376431	3.39399874311542	1.43759483935111	9	c21
coronus	24	1.88832696604995	3.90374661408312	2.01541964803317	12	c24
coronus	24	2.14264700425596	4.79596517456468	2.65331817030872	12	c24
inodorus	12	0.754524162212627	2.01997607144994	1.26545190923732	0	i12
inodorus	12	1.02991061730326	2.76464422213954	1.73473360483628	0	i12
inodorus	12	1.08089095642563	2.9496608503151	1.86876989388948	0	i12
inodorus	12	1.14604405939015	2.63690789011524	1.49086383072509	0	i12
inodorus	12	0.775017533870295	2.56140846029242	1.78639092642213	0	i12
inodorus	12	0.662228001564887	2.10726608276162	1.44503808119674	0	i12
inodorus	15	1.01376664750495	3.05139861701382	2.03763196950887	3	i15
inodorus	15	1.18118731289428	2.58925470681738	1.4080673939231	3	i15
inodorus	15	1.47858279168458	3.02787555798272	1.54929276629814	3	i15
inodorus	15	0.806211656695613	2.70963331150971	1.9034216548141	3	i15
inodorus	15	1.22893421126492	3.56279565254745	2.33386144128253	3	i15
inodorus	15	1.00868199705156	2.56726318531403	1.55858118826248	3	i15
inodorus	18	1.27055844498444	3.46751514990036	2.19695670491592	6	i18
inodorus	18	0.898975807043438	3.7290195658545	2.83004375881107	6	i18
inodorus	18	0.948553813510693	2.25880440299287	1.31025058948217	6	i18
inodorus	18	1.58474411137009	3.77476981552837	2.19002570415827	6	i18
inodorus	18	0.718631441776869	3.18597310545287	2.467341663676	6	i18
inodorus	18	1.1428268565273	2.60378501295675	1.46095815642945	6	i18
inodorus	18	1.26354218767768	3.25757542919666	1.99403324151898	6	i18
inodorus	18	1.39772623944263	3.15896937083355	1.76124313139093	6	i18
inodorus	18	1.7350187221153	2.71347074686226	0.978452024746962	6	i18
inodorus	21	1.50758304576312	3.9289999613721	2.42141691560898	9	i21
inodorus	21	1.50291030794153	3.47363522908499	1.97072492114346	9	i21
inodorus	21	0.632534686715676	3.30562199377222	2.67308730705654	9	i21
inodorus	21	0.979781187690527	3.61351907539746	2.63373788770693	9	i21
inodorus	21	1.23552496456516	4.20834421839283	2.97281925382767	9	i21
inodorus	21	1.27995630847182	3.2256656344702	1.94570932599838	9	i21
inodorus	21	1.52584832955517	3.04623179808912	1.52038346853396	9	i21
inodorus	21	2.46619994088974	4.36629882154956	1.90009888065982	9	i21
inodorus	21	1.36547413638748	3.86679989304812	2.50132575666064	9	i21
inodorus	21	1.16724978753434	3.75943093069485	2.59218114316051	9	i21
inodorus	24	1.42093970276005	5.434730569436	4.01379086667595	12	i24

inodorus	24	1.40498438420027	2.86543543482577	1.4604510506255	12	i24
inodorus	24	1.42686309398702	3.07459111535835	1.64772802137132	12	i24
inodorus	24	1.54359994381425	3.29484134876	1.75124140494575	12	i24
inodorus	24	1.71240908514994	4.97804747965319	3.26563839450325	12	i24
inodorus	24	2.78498951826644	4.07657750784887	1.29158798958244	12	i24
inodorus	24	2.1799099610915	3.54453984917242	1.36462988808092	12	i24
inodorus	24	2.4292540070873	3.1788508508553	0.749596843768005	12	i24
